# Deciphering the interplay between SGLT2 inhibition, CCL4, and angina pectoris: A Mendelian randomization study

**DOI:** 10.1097/MD.0000000000050078

**Published:** 2026-07-31

**Authors:** Yujia Zhang, Yukai Zhao, Zheng Dong, Yuan Gao, Dongmei Wan

**Affiliations:** a The Second Affiliated Hospital of Heilongjiang University of Chinese Medicine, Harbin, Heilongjiang Province, China; b Heilongjiang University of Chinese Medicine, Harbin, Heilongjiang Province, China.

**Keywords:** angina pectoris, C-C motif chemokine ligand 4, inflammatory cytokines, Mendelian randomization, SGLT2 inhibitors

## Abstract

Angina pectoris, a key symptom of coronary artery disease, is closely associated with inflammation. Sodium-glucose cotransporter 2 (SGLT2) inhibitors have shown cardiovascular benefits, but their exact mechanisms, particularly regarding inflammation, are not fully understood. This study used Mendelian Randomization (MR) to investigate whether SGLT2 inhibition reduces angina risk by modulating inflammatory cytokines, especially C-C motif chemokine ligand 4 (CCL4). A two-sample MR approach was employed, using genetic instruments derived from SLC5A2-related eQTLs and HbA1c-associated single nucleotide polymorphisms. Genome-wide association study summary statistics for CCL4 and other inflammatory cytokines were utilized. The primary analysis was conducted using the inverse variance weighted method. SGLT2 inhibition was associated with a significantly reduced risk of angina pectoris (odds ratio [OR] = 0.28, 95% confidence interval [CI]: 0.11–0.70, *P* = .007). Notably, CCL4 was the only inflammatory cytokine linked to both SGLT2 inhibition and angina. SGLT2 inhibition was positively associated with CCL4 levels (β = 1.14, 95% CI: 0.33–1.95, *P* = .006), and CCL4 itself was associated with a higher risk of angina (OR = 1.07, 95% CI: 1.01–1.13, *P* = .022). However, the calculated indirect effect of SGLT2 on angina via CCL4 was negative, indicating that the mediation proportion could not be quantified due to biological complexity. Our study indicates that SGLT2 inhibition may offer protective effects in angina pectoris. The observed association between SGLT2 inhibition and CCL4 suggests a potentially complex inflammatory pathway that warrants further investigation.

## 1. Introduction

Angina pectoris, a common manifestation of coronary artery disease (CAD) characterized by chest discomfort triggered by physical exertion or emotional distress due to myocardial ischemia, remains a substantial global health concern, affecting millions of individuals each year.^[1]^ Despite significant advancements in pharmacological treatments, including β-blockers, calcium channel blockers, Nitrates, and antianginal therapies, the burden of angina pectoris continues to rise.^[2–4]^ The underlying pathophysiology of angina is complex, involving a combination of endothelial dysfunction, inflammation, and metabolic disturbances, which contribute to the narrowing of coronary arteries and impaired myocardial oxygen supply.^[5–7]^ Recent evidence suggests that inflammation plays a pivotal role in the development and progression of CAD, with elevated levels of various cytokines and chemokines being strongly associated with adverse cardiovascular outcomes.^[8,9]^

Sodium-glucose cotransporter 2 (SGLT2) inhibitors, a novel class of diabetes medications, have emerged as promising therapeutic agents in the management of cardiovascular diseases. Initially developed for glycemic control in patients with type 2 diabetes mellitus, SGLT2 inhibitors, such as empagliflozin and canagliflozin, have demonstrated substantial benefits beyond glucose lowering, including reductions in the risk of heart failure, myocardial infarction, and stroke.^[10–12]^ This class of drugs is believed to exert its cardiovascular benefits through mechanisms such as diuresis, blood pressure reduction, and potential modulation of inflammatory pathways.^[13,14]^ However, the exact molecular pathways through which SGLT2 inhibition influences the cardiovascular system, including its effect on inflammatory mediators like chemokines, remain poorly understood.

C-C motif chemokine 4 (CCL4) is a pro-inflammatory cytokine that plays a critical role in recruiting immune cells to sites of inflammation, particularly in atherosclerotic lesions.^[15]^ Elevated CCL4 levels have been implicated in the pathogenesis of CAD, and its association with myocardial ischemia suggests a potential link with angina pectoris.^[16]^ Interestingly, recent studies have shown that SGLT2 inhibitors can modulate inflammatory cytokines, including CCL4,^[17]^ raising the possibility that the anti-atherosclerotic effects of SGLT2 inhibition may, in part, be mediated through the suppression of inflammatory mediators such as CCL4.

Mendelian randomization (MR), an instrumental variable approach using genetic variants as proxies for exposures, offers a robust method for investigating causal relationships in epidemiological research by circumventing limitations such as confounding and reverse causality.^[18,19]^ This study employs a two-sample MR design to explore the interplay between SGLT2 inhibition, CCL4, and angina pectoris, specifically investigating whether SGLT2 inhibition influences the risk of angina pectoris through its effects on inflammatory cytokines, particularly CCL4, and examining the potential mediating role of CCL4. The results will provide valuable insights into the mechanisms by which SGLT2 inhibition may reduce cardiovascular risk, especially in the context of angina pectoris, and underscore the potential role of inflammatory pathways in these processes.

## 2. Methods

### 2.1. Study design

**Figure 1.**
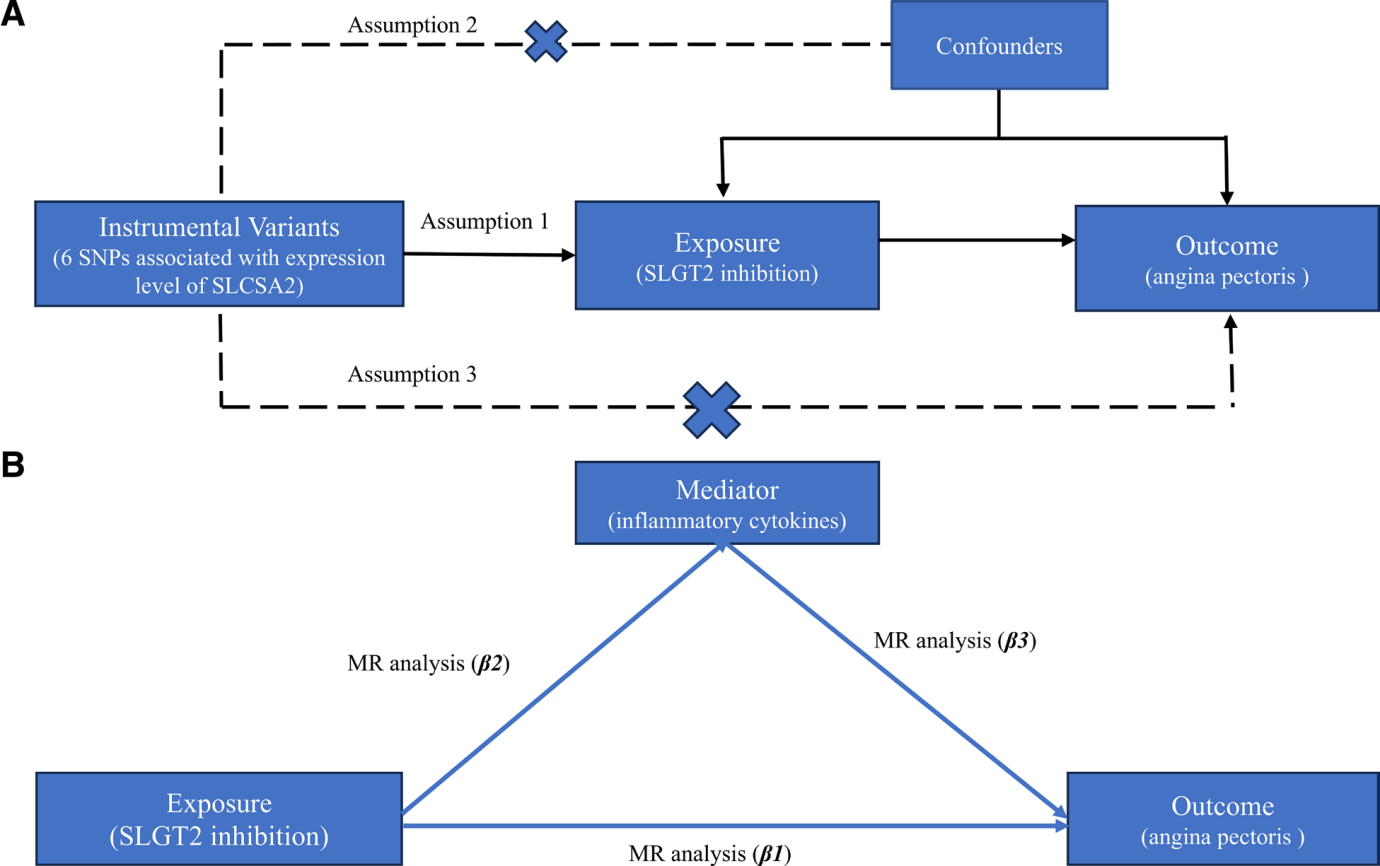
Study design flowchart of the Mendelian randomization study. (A) Overview of the Mendelian randomization. Assumption 1: the IVs must be strongly associated with the exposure; Assumption 2: the IVs should be independent of the potential confounding factors of the exposure-outcome association; Assumption 3: the IVs should not be directly linked to outcomes. (B) The framework of the two-step method of Mendelian randomization. Total effect = β1; Mediation effect = β2×β3; Direct effect = β1-β2×β3; Proportion mediated = (β2×β3)/β1. IV, instrumental variable; SGLT2, sodium-glucose cotransporter 2; SNP, single nucleotide polymorphism.

This study employed a two-sample MR design to investigate the causal relationships between SGLT2 inhibition, inflammatory cytokines, and angina pectoris (Fig. 1A). MR analyses were based on 3 core assumptions: (1) the instrumental variables (IVs) are strongly associated with the exposure, (2) the IVs are independent of confounders, and (3) the IVs influence the outcome only through the exposure. We first evaluated the causal effect of SGLT2 inhibition on angina pectoris and then used a two-step MR approach to explore the mediating role of inflammatory cytokines (Fig. 1B). This study follows the STROBE-MR guidelines^[20]^ with detailed methods outlined in Table S1, Supplemental Digital Content 1. This study uses publicly available GWAS summary data, all of which have received ethical approval. As no individual-level data is used, no additional ethical review is required.

### 2.2. Genetic instruments for SGLT2 inhibition

The genetic instruments for SGLT2 inhibition were selected through a four-step process. First, genetic variants associated with the mRNA expression level of the SLC5A2 gene, the target gene for SGLT2 inhibition, were identified using data from the Genotype-Tissue Expression project^[21]^ and the eQTLGen Consortium.^[22]^ Second, the associations between SLC5A2 variants and glycated hemoglobin levels, indicative of the glucose-lowering effects of SGLT2 inhibition, were evaluated. Variants significantly associated with HbA1c (*P* < 1 × 10^−4^) were selected based on data from nondiabetic individuals of European ancestry in the UK Biobank (n = 344,182). Third, genetic colocalization analysis was conducted to confirm that SLC5A2 expression and HbA1c levels shared the same causal variant, with a posterior probability > 70% considered as evidence of colocalization.^[23]^ Finally, linkage disequilibrium (LD) clumping was performed using an r^2^ < 0.8 threshold to remove highly correlated variants.^[24]^ The strength of each variant as an instrumental variable was assessed using F-statistics. Following these rigorous steps, 6 genetic variants robustly associated with SGLT2 inhibition were identified as instruments for Mendelian Randomization analysis (see Table S2, Supplemental Digital Content 2).

### 2.3. Genetic instruments for inflammatory cytokines

The GWAS data on 91 inflammatory cytokines were obtained from 14,824 individuals of European descent across 11 cohorts (accession numbers GCST90274758 to GCST90274848). Genome-wide pQTL mapping was performed for plasma proteins measured using the Olink Target Inflammation panel.^[25]^ More details are available in Table S3, Supplemental Digital Content 3. The GWAS summary data can be accessed via: http://ftp.ebi.ac.uk/pub/databases/gwas/summary_statistics.

### 2.4. Genetic instruments for angina pectoris

The angina pectoris data used in this study were obtained from the Finnish database (FinnGen Biobank). The data include 18,168 cases and 187,840 controls, with a total of 16,380,426 single nucleotide polymorphisms (SNPs).^[26]^

### 2.5. The selection of IVs

SNPs significantly associated with inflammatory cytokines were identified using a *P*-value threshold of < 1e^-6^. To reduce potential pleiotropy, those with high LD were excluded based on an LD coefficient threshold (kb = 10,000, r^2^ = 0.001). IVs were selected by retaining SNPs with F-statistics > 10 to minimize weak instrument bias. To avoid allele ambiguity between exposure and outcome traits, palindromic SNPs were excluded.

### 2.6. Statistical analyses

In the primary MR analyses exploring the causal relationship between SGLT2 inhibition and angina pectoris, 5 MR methods were applied: MR Egger, weighted median, inverse variance weighted (IVW), simple mode, and weighted mode. The IVW method, known for its high statistical power when all genetic variations are valid IVs, was used as the primary approach to estimate causal effects. Heterogeneity was assessed using Cochrane’s Q-test (*P* > .05 indicating no significant heterogeneity), and horizontal pleiotropy was evaluated with the MR-Egger intercept test.

A two-step MR approach was used to identify inflammatory cytokines mediating the effects of SGLT2 inhibition on angina pectoris. In the first step, MR analyses with the IVW method were conducted to assess the causal effects of 91 inflammatory cytokines on angina pectoris. Inflammatory cytokines that met the significance threshold (*P* < .05) were carried into the second step, where SGLT2 inhibition on their causal effects were examined. The MR estimates for SGLT2 inhibition on angina pectoris, SGLT2 inhibition on each inflammatory cytokine, and each inflammatory cytokine on angina pectoris were denoted as β1, β2, and β3, respectively. For inflammatory cytokines with significant β2 and β3 values, mediation analysis was performed to determine whether they mediate the effect of SGLT2 inhibition on angina pectoris risk. The indirect effect, representing SGLT2 inhibition ‘s impact on angina pectoris via the cytokines, was calculated using the “Product of coefficients” method (β2 × β3).^[27]^ The proportion of mediation was estimated as the ratio of the indirect effect to the total effect (β2×β3/β1). 95% confidence intervals (CIs) were computed using the delta method. As a sensitivity analysis, false discovery rate (FDR) correction using the Benjamini–Hochberg method was applied to account for multiple testing across the 91 inflammatory cytokines.

All analyses and data visualization were performed using R (version 4.3.2). Univariable MR analysis was conducted with the R packages “TwoSampleMR” and “MendelianRandomization.”

## 3. Results

### 3.1. Effect of SGLT2 inhibition on angina pectoris

**Figure 2.**
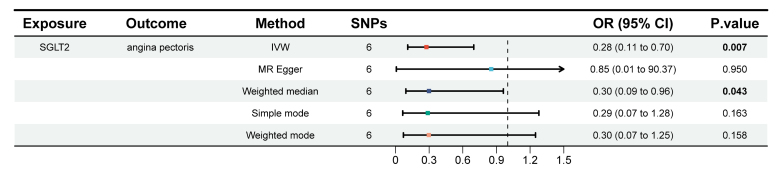
The causal effect of SGLT2 inhibition on angina pectoris. IVW = inverse variance weighted; OR = odds ratio; CI = confidence interval; SGLT2 = sodium-glucose cotransporter.

In total, 6 independent SNPs were selected as genetic instruments for SGLT2 inhibition and the F statistics for all SNPs were >10 (Table S2, Supplemental Digital Content 2). In MR analysis, we found that SGLT2 inhibition was associated with reduced risk of angina pectoris (odds ratio [OR] = 0.28 [95% CI 0.11, 0.70], *P* = .007) (Table 1, Figure 2). There was no heterogeneity between instruments for the effect of SGLT2 inhibition on angina pectoris (Q_Ivw_ = 0.752, P_Ivw_ = 0.980; Q_MR-Egger_ = 0.518, *P* = .972), and no horizontal pleiotropy was detected (Egger intercept = −0.014, *P* = .654)(Table 1).

**Table 1:** MR estimates of the effect of SGLT2 inhibition on angina pectoris.

Exposure	Outcome	Method	OR (95% CI)	P	Q statistic	*P*-heterogeneity	Egger intercept	P-intercept
SGLT2	angina pectoris	IVW	0.28 (0.11,0.70)	0.007	0.752	0.980		
		MR Egger	0.85 (0.01,90.37)	0.950	0.518	0.972	-0.014	0.654
		Weighted median	0.30 (0.09,0.96)	0.043				
		Simple mode	0.29 (0.07,1.28)	0.163				
		Weighted mode	0.30 (0.07,1.25)	0.158				

Odds ratio (OR), 95% confidence interval (CI), and P values were calculated for the respective method of MR analysis. *P* < .05 was considered significant. IVW = inverse-variance weighted, *P*-heterogeneity, *P* value for heterogeneity test; *P*-intercept, *P* value for the intercept of MR-Egger regression.

### 3.2. Mediation MR of SGLT2 inhibition, inflammatory cytokines, and angina pectoris

**Figure 3.**
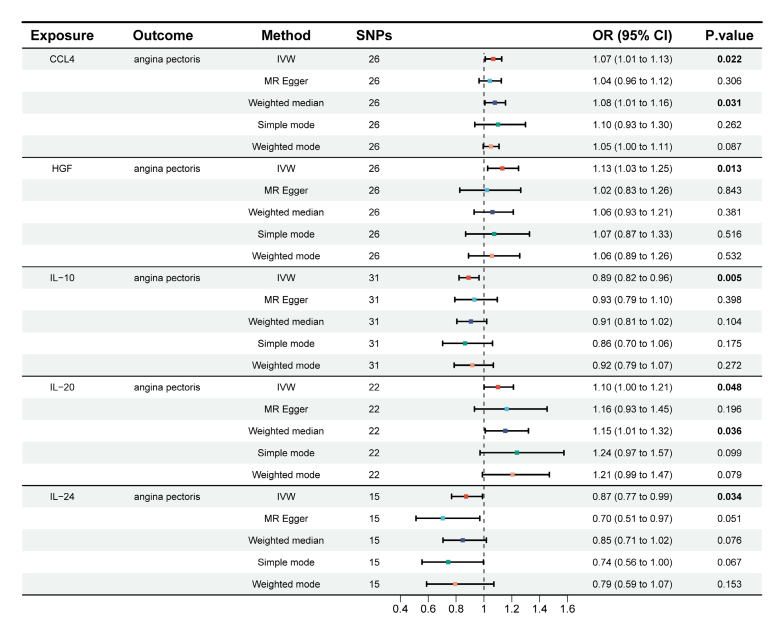
The causal effect of inflammatory cytokines on angina pectoris. CCL4 = C-C motif chemokine 4, CI = confidence interval, HGF = hepatocyte growth factor, IL10 = interleukin 10, IL20 = interleukin 20, IL24 = interleukin 24, IVW = inverse variance weighted, OR = odds ratio.

We estimated the effect of 91 inflammatory cytokines on angina pectoris and observed that 5 inflammatory cytokines were nominally associated with angina pectoris (*P* < .05) (Fig. 3). Additional FDR correction was performed as a sensitivity analysis; however, none of these associations remained statistically significant after correction. We found that CCL4 levels (OR = 1.07 [95% CI 1.01, 1.13], *P* = .022), hepatocyte growth factor (HGF) levels (OR = 1.13 [95% CI 1.03, 1.25], *P* = .013), and interleukin-20 (IL-20) levels (OR = 1.10 [95% CI 1.00, 1.21], *P* = .048) had positive association with angina pectoris, and interleukin-10 (IL-10) levels (OR = 0.89 [95% CI 0.82, 0.96], *P* = .005), interleukin-24 (IL-24) levels (OR = 0.87 [95% CI 0.77, 0.99], *P* = .034) had negative association with angina pectoris. No evidence of significant heterogeneity or horizontal pleiotropy was observed across the analyses (all *P* > .05) (Table 2).

**Table 2:** Heterogeneity and horizontal pleiotropy of 5 inflammatory cytokines and angina pectoris.

Exposure	Outcome	Method	Q statistic	*P*-heterogeneity	Egger intercept	P-intercept
CCL4	angina pectoris	IVW	35.858	.074		
		MR Egger	34.713	.073	0.007	0.382
HGF	angina pectoris	IVW	27.239	.344		
		MR Egger	26.015	.352	0.011	0.299
IL-10	angina pectoris	IVW	36.644	.188		
		MR Egger	36.156	.169	-0.005	0.536
IL-20	angina pectoris	IVW	13.043	.907		
		MR Egger	12.752	.888	-0.008	0.595
IL-24	angina pectoris	IVW	11.326	.660		
		MR Egger	9.316	.749	0.022	0.180

CCL4 = C-C motif chemokine 4, IL10 = interleukin 10, IL20 = interleukin 20, IL24 = interleukin 24, HGF = hepatocyte growth factor, IVW = inverse variance weighted.

**Figure 4.**
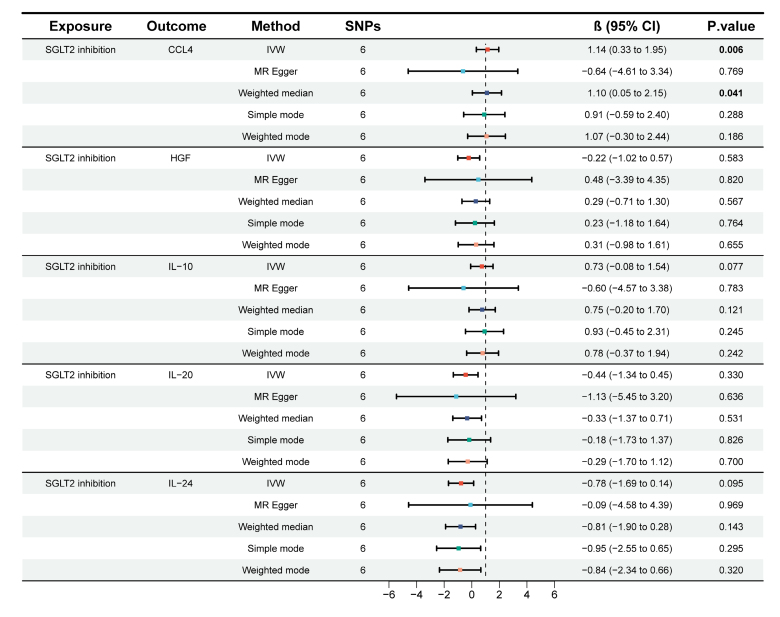
The causal effect of SGLT2 inhibition on inflammatory cytokines. SGLT2 = sodium-glucose cotransporter; CCL4 = C-C motif chemokine 4, CI = confidence interval, HGF = hepatocyte growth factor, IL10 = interleukin 10, IL20 = interleukin 20, IL24 = interleukin 24, IVW = inverse variance weighted, OR = odds ratio.

We further examined the effects of the 5 inflammatory cytokines significantly associated with angina pectoris and identified that only 1 cytokine (CCL4) showed a significant association with SGLT2 inhibition (*P* < .05). We found that SGLT2 inhibition had positive association with CCL4 (β = 1.14 [95% CI 0.33, 1.95], *P* = .006), but little evidence to support association with SGLT2 inhibition and HGF, IL-10, IL-20, and IL-24 (Fig. 4). The Q statistics and P values were not significant (*P* values from 0.416 to 0.924), which implied no evidence of heterogeneity. The pleiotropy test using the MR-Egger intercept term showed that P values of the intercepts varied from 0.421 to 0.776, which meant little evidence of directional pleiotropy (Table S4, Supplemental Digital Content 4).

Two-step MR analysis was conducted to assess whether the association between SGLT2 inhibition and the risk of angina pectoris was mediated through CCL4. The study revealed that SGLT2 was negatively associated with the risk of angina pectoris (β1 = −1.285 [95% CI −2.212, −0.360]), positively associated with CCL4 levels (β2 = 1.143 [95% CI 0.332, 1.953]), and CCL4 levels were positively associated with the risk of angina pectoris (β3 = 0.065 [95% CI 0.009, 0.120]). The calculated indirect effect of SGLT2 on angina pectoris through CCL4 was negative, indicating that the mediation proportion could not be quantified due to the lack of biological interpretability.

## 4. Discussion

Our study provides evidence that SGLT2 inhibition is associated with a reduced risk of angina pectoris. Specifically, we found that SGLT2 inhibition was inversely associated with the risk of angina (odds ratio [OR] = 0.28 [95% CI 0.11, 0.70], *P* = .007), indicating a protective effect. Additionally, we observed that SGLT2 inhibition was positively correlated with the inflammatory cytokine CCL4 (β = 1.14 [95% CI 0.33, 1.95], *P* = .006). In contrast, CCL4 itself was positively associated with an increased risk of angina pectoris (OR = 1.07 [95% CI 1.01, 1.13], *P* = .022). This suggests that the observed relationship between SGLT2 inhibition and reduced risk of angina may, in part, involve modulation of CCL4 levels. However, while the positive correlation between SGLT2 inhibition and CCL4 was significant, the mediation effect via CCL4 could not be quantified due to the complex nature of the biological mechanisms underlying this association.

The main findings of our study suggest that while SGLT2 inhibition may provide a protective effect in angina pectoris, CCL4 may represent a potentially complex inflammatory pathway associated with this relationship. SGLT2 inhibitors are well-documented for their cardiovascular benefits, particularly in reducing the risk of heart failure and improving myocardial function.^[28,29]^ However, protective effects can be counteracted by inflammatory pathways, specifically CCL4-mediated immune responses. CCL4 is a potent chemokine involved in the recruitment of immune cells to sites of inflammation, and its role in the pathogenesis of atherosclerosis and myocardial ischemia is well documented.^[30–32]^ This suggests that while SGLT2 inhibitors hold immense therapeutic potential, their pro-inflammatory effects, particularly through CCL4, may diminish their overall benefits. This interaction highlights the importance of considering complex inflammatory mechanisms when using SGLT2 inhibitors in clinical practice, as their effects may not be purely protective. Clinically, these findings indicate that patients with elevated inflammatory status or heart failure may experience attenuated cardiovascular benefits from SGLT2 inhibition.^[33,34]^ Awareness of this potential interaction can help guide risk stratification and optimize therapeutic decision-making in real-world populations.^[35]^

Our study also emphasizes the pivotal role of inflammation in cardiovascular diseases like angina pectoris. In particular, CCL4’s involvement in immune cell recruitment and its established role in atherosclerosis and cardiovascular events, such as myocardial infarction and stroke, makes it a potential target for modulating the effects of SGLT2 inhibitors. Interestingly, our results showed that SGLT2 inhibition was associated with an increase in CCL4 levels, which contrasts with the known effect of SGLT2 inhibitors in reducing systemic inflammation and lowering inflammatory markers.^[36–38]^ This unexpected elevation in CCL4 suggests that the impact of SGLT2 inhibitors on cardiovascular disease may be more nuanced than previously thought, possibly involving a complex interplay of both protective and pro-inflammatory effects. Consequently, further studies are needed to clarify the biological significance of the observed association between SGLT2 inhibition and CCL4. It should also be noted that the observed associations did not remain statistically significant after FDR correction and therefore require cautious interpretation.

Given the potential for CCL4 to counteract the protective effects of SGLT2 inhibition, we suggest that adjunctive therapies targeting CCL4 may be a promising avenue for enhancing the cardiovascular benefits of SGLT2 inhibitors. Although direct studies on CCL4 antagonists or inhibitors are limited, recent research has explored the broader potential of targeting CCL4 in modulating inflammatory processes and atherosclerosis.^[30,39]^ Combining SGLT2 inhibitors with CCL4-targeting agents could potentially amplify the anti-atherosclerotic and anti-inflammatory effects, thereby improving outcomes for patients with angina pectoris and other cardiovascular diseases. Additionally, the development of novel SGLT2 inhibitors that do not induce an elevation in CCL4 could further optimize treatment strategies, particularly for patients who are at high risk for cardiovascular events.

Moreover, Our results further support the notion that inflammation plays a key role in the progression of heart disease, particularly angina pectoris. We identified specific inflammatory cytokines, including IL-10, IL-20, IL-24, and NGF, as key mediators associated with angina pectoris. These findings are consistent with existing literature showing that interleukins such as IL-10, and neurotrophic factors like NGF, are implicated in the development of atherosclerosis and angina pectoris.^[40–42]^ Interestingly, while these cytokines were linked to the pathophysiology of angina, no direct relationship was found between these inflammatory markers and SGLT2 inhibition. This highlights an important aspect of our findings: while SGLT2 inhibitors are well-documented for their cardiovascular benefits, the role of specific inflammatory mediators, such as IL-10 and NGF, in heart disease pathogenesis may operate independently of SGLT2 inhibition. Targeting these inflammatory pathways could provide additional therapeutic benefits in managing cardiovascular diseases, although further research is needed to fully understand how these cytokines interact with treatment regimens, including those involving SGLT2 inhibitors.

This study is the first to use MR to investigate the causal relationship between SGLT2 inhibition, inflammatory cytokines, and angina, offering a novel approach to understanding the mechanisms underlying angina pectoris risk reduction. However, there are several limitations to consider. First, the use of publicly available GWAS summary data may not fully capture genetic diversity across populations, potentially limiting generalizability to non-European cohorts. Second, MR studies rely on the assumption that the genetic variants used as instruments are not affected by pleiotropy, but we cannot completely rule out the possibility of horizontal pleiotropy, where genetic variants may influence outcomes through pathways other than the proposed mechanism. Third, the limited number of genetic instruments for SGLT2 inhibition (n = 6) may reduce the statistical power of MR-Egger and mode-based sensitivity analyses; therefore, these results should be interpreted as supportive rather than definitive evidence. Fourth, the study lacks individual-level clinical data, such as treatment regimens, comorbidities, and lifestyle factors, which may influence real-world applicability. Finally, although several inflammatory cytokines showed nominal associations with angina pectoris, none remained significant after FDR correction for multiple testing. Therefore, these findings should be considered exploratory and require further validation in independent studies.

In conclusion, Genetically proxied SGLT2 inhibition was associated with a reduced risk of angina pectoris. Among the inflammatory cytokines examined, CCL4 was the only cytokine associated with both SGLT2 inhibition and angina pectoris. Although the observed direction of effects does not support a conventional mediation pathway, it may indicate the presence of a potential counteracting inflammatory mechanism involving CCL4. Further mechanistic and clinical studies are warranted to clarify the biological significance of this association.

## Author contributions

**Formal analysis:** Yujia Zhang, Yuan Gao.

**Methodology:** Yujia Zhang, Zheng Dong.

**Resources:** Yujia Zhang, Yukai Zhao.

**Writing – original draft:** Yujia Zhang, Zheng Dong.

**Writing – review & editing:** Yujia Zhang, Yukai Zhao, Dongmei Wan.

**Investigation:** Yukai Zhao.

**Software:** Yukai Zhao, Zheng Dong, Yuan Gao.

**Validation:** Yukai Zhao, Zheng Dong.

**Conceptualization:** Zheng Dong, Yuan Gao.

**Supervision:** Zheng Dong.

**Visualization:** Zheng Dong, Dongmei Wan.

**Data curation:** Yuan Gao.

**Funding acquisition:** Dongmei Wan.

**Project administration:** Dongmei Wan.








